# The *Comrades Marathon:* a narrative review of physiological responses and health implications in the world’s oldest ultra-marathon

**DOI:** 10.3389/fphys.2026.1811456

**Published:** 2026-05-18

**Authors:** Beat Knechtle, Volker Scheer, Pantelis T. Nikolaidis, Mabliny Thuany, Daniela Chlíbková, Pedro Forte, Luciano Bernardes Leite, Katja Weiss, Thomas Rosemann, Sasa Duric

**Affiliations:** 1 Medbase St. Gallen Am Vadianplatz, St. Gallen, Switzerland; 2 Institute of Primary Care, University Hospital Zurich, Zurich, Switzerland; 3 Ultra Sports Science Foundation, Pierre-Bénite, Auvergne-Rhône-Alpes, France; 4 School of Health and Caring Sciences, University of West Attica, Athens, Greece; 5 Department of Sports, State University of Pará, Belém, Brazil; 6 Centre of Sports Activities, Brno University of Technology, Brno, Czechia; 7 Department of Sports, Higher Institute of Educational Sciences of the Douro, Penafiel, Portugal; 8 Department of Sports Sciences, Instituto Politécnico de Bragança, Bragança, Portugal; 9 Research Center for Active Living and Wellbeing (Livewell), Instituto Politécnico de Bragança, Bragança, Portugal; 10 Department of Physical Education, Federal University of Viçosa, Viçosa, Brazil; 11 American University of the Middle East, Egaila, Kuwait

**Keywords:** cardiac function, fluid balance, metabolism, skeletal muscle damage, ultra-endurance

## Abstract

**Introduction:**

The *Comrades Marathon* is the world’s oldest and largest ultra-marathon, held annually between Durban and Pietermaritzburg, South Africa, since 1921. As a nationally televised event with substantial participation, it provides a unique natural laboratory for studying the physiological demands of prolonged, high-intensity endurance running. This narrative review synthesizes current evidence on physiological responses, performance trends, and health implications associated with participation in the *Comrades Marathon*.

**Methods:**

A systematic search of EBSCO, PubMed, SciELO, and Web of Science identified studies published up to December 2025. Of 116 records retrieved, 42 publications (field studies and case reports) met eligibility criteria and focused specifically on the *Comrades Marathon*.

**Results:**

Women represented 4.2% of finishers, with female participation beginning in 1975. Growth in finishers during the 1970s was driven primarily by men aged 30–59 years. Men were consistently faster than women across all age groups, although the sex performance gap narrowed over time and overall performance improved. Peak performance occurred at ∼29.9 years in men and ∼36.0 years in women. Medical encounters occurred at rates up to 20 per 1000 starters. The most affected organ systems were fluid/electrolyte (8.8%; 8.3–9.4), central nervous system (4.0%; 3.7–4.5), and gastrointestinal (2.9%; 2.6–3.2). Dehydration (7.5%; 7.0–8.1) and exercise-associated muscle cramping (3.2%; 2.9–3.6) were the most common diagnoses. Exercise-associated hyponatremia was uncommon (<2%), whereas hypernatremia was substantially more prevalent. Early race editions reported isolated deaths, likely due to ischemic heart disease. Cardiac biomarker elevations and echocardiographic changes observed post-race were transient. The race induced muscle inflammation without major knee MRI abnormalities. In the 1970s, cases of acute kidney injury and renal failure occasionally required dialysis. Faster, well-trained runners showed higher rates of post-race upper respiratory tract infection than slower, less-trained runners.

**Conclusions:**

Participation in the *Comrades Marathon* is associated with well-characterized post-race physiological disturbances. Notably, the event demonstrates an unusually high prevalence of acute kidney injury and hypernatremia compared with other ultra-endurance races, underscoring the substantial renal and fluid–electrolyte stress imposed by prolonged, high-intensity running in challenging environmental conditions. These insights can inform individualized hydration strategies, targeted race preparation, and optimized medical support for ultra-endurance athletes.

**Systematic review registration:**

https://www.crd.york.ac.uk/PROSPERO/view/CRD420251252644, identifier CRD420251252644.

## Introduction

The *Comrades Marathon* is a road-running ultra-marathon contested between the South African cities of Durban and Pietermaritzburg since 1921 (Labuschagne, 2009). Conceived by World War I veteran Vic Clapham to honor South African soldiers who died in the war, it has evolved into the world’s most traditional and largest ultra-marathon and is broadcast nationally in its entirety (The comrades marathon).

A defining feature of the *Comrades Marathon* is its annually alternating direction, which effectively creates two distinct races. The Up Run (Durban to Pietermaritzburg) and the Down Run (Pietermaritzburg to Durban) share a common ethos and comparable distance, yet impose markedly different physiological, biomechanical, and psychological demands. The Up Run includes ∼1,800–1,900 m of total ascent and ~1,200 m of descent, characterized by long, sustained climbs. In contrast, the Down Run features ~1,200 m of ascent and 1,800–1,900 m of descent, with extended downhill sections that generate substantial eccentric loading. This alternating course direction provides a unique natural experiment for examining the differential effects of concentric versus eccentric muscle loading on skeletal muscle damage over time (Milledge, 1977). The race traditionally begins at the town hall of the host city, while varying finish venues in the destination city result in route lengths ranging from 86 to 92 km (The comrades marathon).

Women were officially permitted to enter the race from 1975 onward, according to the Comrades Marathon Association and Noakes’ *Lore of Running*. However, the first female participation dates back to Frances Hayward in 1923, albeit unofficially (The comrades marathon). While some dataset-specific analyzes report the first female finishers appearing in 1978 (Nikolaidis et al., 2021), historical records from the Comrades Marathon Association confirm that official entry opened in 1975 (Merrett, 2007; Jack, 2015).

Scientific interest in the *Comrades Marathon* also has a long history. The first studies emerged in the 1970s (Dancaster and Whereat, 1971; Olivier et al., 1978; MacSearraigh et al., 1979; McKechnie et al., 1982) and 1980s (McKechnie et al., 1982), focusing on fluid metabolism (Dancaster and Whereat, 1971), kidney injury—including acute renal failure (MacSearraigh et al., 1979)—and potential cardiac damage (Olivier et al., 1978; McKechnie et al., 1982). Notably, Noakes’ 1981 and 1982 studies went beyond describing ‘metabolic responses’; they challenged prevailing hydration guidelines and contributed to the early identification of exercise-associated hyponatremia (EAH) (McKechnie et al., 1982).

As participation expanded to ∼20,000 participants per year/event, the number of medical emergencies increased, prompting the development of the SAFER (Strategies to reduce Adverse medical events For the ExerciseR) studies. The foundational paper by Schwabe et al. initiated a comprehensive research program (Schwabe et al., 2014), with subsequent SAFER studies (Brill et al., 2023; Slabber et al., 2023; Havenga et al., 2024; Macmillan et al., 2024; Sewry et al., 2024) focusing primarily on medical encounters (MEs) (Leppan et al., 2023; Sewry et al., 2024) and serious and life-threatening medical encounters (SLMEs) (Sewry et al., 2024). Within this framework, the HEAT (Healthy Environments for AthleTes) project examined the impact of environmental conditions on athlete health and performance during endurance events (Havenga et al., 2024). Additional research explored the strategic development of virtual sports during the COVID-19 pandemic (Woyo and Nyamandi, 2022).

Participation in the *Comrades Marathon* is subject to several restrictions. The time limit for the ∼90 km course is twelve hours (The comrades marathon). Due to its mountainous terrain, the race is unsuitable for athletes with certain disabilities, such as wheelchair athletes (Maralack, 2016). Women (Labuschagne, 2009; Naidoo, 2012) and Black runners (Merrett, 2007; Labuschagne, 2009) were historically excluded but are now fully integrated into the event. Today, the entry limit of 20,000 participants is often reached within hours of registration opening (The comrades marathon).

Despite more than a century of competition and numerous isolated studies, no comprehensive synthesis exists that integrates performance trends, demographic shifts, medical encounter epidemiology, and multi-system physiological responses specific to the *Comrades Marathon*. Key gaps include the absence of a unified interpretation of renal and electrolyte disturbances, limited contextualization of *Comrades Marathon* relative to other ultra-marathon events, and a lack of evidence-based implications for hydration, pacing, and medical support. This review addresses these gaps by consolidating findings from 42 specific publications from the *Comrades Marathon* to provide the first integrated physiological and clinical profile of this uniquely demanding ultra-marathon.

Therefore, in this narrative review, we synthesize the existing scientific literature on the *Comrades Marathon* to provide a comprehensive overview of physiological responses, performance trends, and associated health risks. These insights may assist athletes, coaches, and medical teams in preparing effectively for this unique ultra-marathon.

## Methods

### Search strategy, eligibility criteria, and data extraction

This narrative review searched for scientific evidence related to the *Comrades Marathon* using four electronic databases: PubMed, EBSCO, Web of Science, and SciELO (Sukhera, 2022). All articles published up to December 2025 were considered. These databases were selected for their broad coverage of biomedical, sports science, and interdisciplinary research, ensuring comprehensive retrieval of relevant literature. The search strategy combined Medical Subject Headings (MeSH) with free-text keywords. In PubMed, the field tag *[tiab]* was used to restrict free-text terms to titles and abstracts, while MeSH terms captured indexed subject headings. No filters were applied for language, sex, or study design to maximize sensitivity. The full Boolean search string used in PubMed was: (“Comrades Marathon”[tiab] OR “Comrades ultramarathon”[tiab] OR “Comrades race”[tiab] OR “ultramarathon”[MeSH] OR ultramarathon*[tiab] OR “ultra marathon*”[tiab] OR “ultra-endurance”[tiab] OR “ultra endurance”[tiab] OR “endurance running”[MeSH] OR “endurance run*”[tiab] OR “long-distance running”[MeSH] OR “long distance run*”[tiab]) AND (participation[tiab] OR “race participation”[tiab] OR finishers[tiab] OR DNF[tiab] OR performance[tiab] OR “race performance”[tiab] OR “finish time”[tiab] OR age[MeSH] OR age[tiab] OR “peak performance age”[tiab] OR “body weight”[MeSH] OR “body mass”[tiab] OR “weight change”[tiab] OR “cardiovascular system”[MeSH] OR cardiovascular[tiab] OR “heart rate”[tiab] OR “musculoskeletal system”[MeSH] OR musculoskeletal[tiab] OR “muscle damage”[tiab] OR nutrition[MeSH] OR nutrition[tiab] OR “sports nutrition”[tiab] OR “body fluids”[MeSH] OR hydration[tiab] OR dehydration[tiab] OR “oxidative stress”[MeSH] OR “oxidative stress”[tiab] OR “immune system”[MeSH] OR immune[tiab] OR cytokine*[tiab]).

Eligible studies included original research conducted on athletes of any sex, age group, or competitive level participating in the *Comrades Marathon*, and addressing topics such as physiological and pathophysiological aspects, nutrition, influence on systems, pacing strategy, performance, or participant characteristics. Non-original publications—including grey literature, book chapters, editorials, and conference proceedings—were excluded. No restrictions were applied regarding publication date or language. Reference lists of included studies were also screened to identify additional relevant publications.

The identification and screening processes were conducted by the lead author (BK) in collaboration with co-authors (SD and MT). All records retrieved through the search strategy were compiled by the lead author, and duplicate entries were removed independently by two authors (BK and MT). After removing duplicates, the remaining records were screened by title and abstract. Studies considered potentially eligible were then assessed through full-text reading.

Eligibility criteria were applied at the full-text stage. The population of interest included human participants of both biological sexes; animal and *in vitro* studies were excluded. The intervention of interest was participation in the *Comrades Marathon*, and studies involving other populations or other ultra-marathon events were excluded. For the comparator, studies were required to include assessments conducted pre-race, during the race, or post-race; studies lacking clearly defined assessment time points were excluded. Eligible outcomes included variables related to participation, performance, age of peak performance, changes in body mass, cardiovascular and musculoskeletal responses, nutrition and fluid metabolism, oxidative stress, and immune system responses. Studies addressing outcomes unrelated to physiological, performance, or participation aspects were excluded. Only original research articles and case reports published in peer-reviewed journals were included, while reviews, systematic reviews, meta-analyzes, editorials, letters, conference abstracts, theses, dissertations, study protocols, and non–peer-reviewed publications were excluded.

Data extraction was performed independently by two authors (BK and MT). Extracted information included: (1) authors; (2) year of publication; (3) study design; (4) sample characteristics; (5) running or race characteristics; (6) variables assessed; (7) main results; and (8) health-associated factors. Given the substantial heterogeneity in study designs, outcomes, and measurement approaches, quantitative synthesis was not feasible. Instead, studies were grouped according to their primary domain of investigation, and results were narratively synthesized within these thematic categories.

### Study appraisal and synthesis of evidence

Because this review followed a narrative approach, no formal risk-of-bias assessment or standardized methodological quality appraisal was undertaken. Our objective was to provide a comprehensive and integrative overview of the scientific literature on the *Comrades Marathon* rather than to conduct a structured evaluation of study quality. The included evidence was organized into key thematic domains—participation, performance, and physiological responses—and synthesized descriptively. Interpretations were developed within the broader context of ultra-endurance research, without the use of a predefined qualitative or quantitative synthesis protocol.

## Results

### Literature search

A total of 116 publications were identified across the four databases: 36 in PubMed, 27 in EBSCO, 47 in Web of Science, and 6 in SciELO. After removing duplicates and excluding articles unrelated to the topic during title and abstract screening, 42 publications—comprising field studies and case reports—met the eligibility criteria and were included in this review. All included studies focused specifically on the *Comrades Marathon*.

Based on the extracted findings, the results are presented in three thematic sections: (1) participation and performance trends, (2) physiological responses, and (3) medical complications. This structure reflects the major domains represented in the available literature and supports an integrated interpretation of the event’s unique demands.

### Section 1

In this section, we synthesize the available evidence on participation patterns, long-term performance trends, and the age of peak performance in the *Comrades Marathon*.

### Participation and performance trends

#### Participation trends

Long-term analyzes of the *Comrades Marathon* demonstrate substantial shifts in participation across nearly a century of competition. Until 1978, the race was contested exclusively by men, and all participation growth prior to this date — including the marked expansion during the 1970s — reflects increases among male runners, particularly those aged 30–39, 40–49, and 50–59 years (Nikolaidis et al., 2021). This period saw a pronounced broadening of the male participant base, contributing to a steady rise in total field size.

Women first appeared as official finishers in 1978, but their numbers remained extremely low throughout the 1980s and early 1990s, with annual fields typically ranging from 5 to 35 runners (Nikolaidis et al., 2021). Despite the overall growth of the event during these decades, female participation did not yet contribute meaningfully to field expansion. A sustained and substantial rise in female participation emerged only from the mid-1990s onward, when the number of women entering and finishing the race began to increase sharply (Nikolaidis and Knechtle, 2019). Across the full historical dataset, women represented ∼4.2% of all participants (Nikolaidis et al., 2021), reflecting the long period of male dominance in the event’s demographic profile.

#### Performance trends

Performance patterns also evolved markedly over time. Across the modern dataset, both women and men demonstrated progressive improvements in race times between 1994 and 2017 (Nikolaidis and Knechtle, 2019). Although male runners consistently outperformed female runners across all age groups throughout the historical record (Nikolaidis et al., 2021), the sex performance gap narrowed steadily over the decades. Early analyzes indicated that women were typically ∼10–20% slower than men depending on age group and race year, whereas by the 2000s and 2010s the gap had contracted to single-digit percentage differences in several age categories (Nikolaidis and Knechtle, 2019). This reduction reflects a clear convergence in performance levels across sexes during the modern era.

Nationality-based analyzes revealed substantial variation in performance outcomes. Russian runners recorded the fastest average race times, whereas Indian runners achieved the slowest performances within the same period (Nikolaidis and Knechtle, 2019). When examining the entire field, average running speed for both sexes remained relatively stable over time, suggesting that mass-participation dynamics did not substantially alter mean performance levels. In contrast, the annual top five male and female finishers showed clear improvements in running speed, indicating that elite competitors continued to advance the competitive frontier even as overall field characteristics stabilized (Nikolaidis et al., 2021).

A complementary dataset comprising 235,467 finishers (40,211 women; 195,256 men) from 1994–2017 further confirmed an overall improvement in performance during this period (Nikolaidis and Knechtle, 2019), reinforcing the pattern of enhanced competitiveness in the modern era.

### Age of peak performance in the *Comrades Marathon*

Analyses of 202,370 race finishers (34,090 women; 168,280 men) from 1994 to 2015 revealed clear sex-specific differences in the age of peak performance. Performance was examined in 1-year age intervals. When considering all finishers, men achieved their fastest mean race times at ∼29.89 years, approximately six years earlier than women, who peaked at ∼35.96 years. However, when the analysis was restricted to the fastest competitors within each 1-year interval, this pattern reversed: elite men reached peak performance at ∼36.38 years, roughly four years later than elite women, who peaked at ∼32.75 years (Knechtle and Nikolaidis, 2017).

Across the broader field, the average age of finishers increased steadily between 1994 and 2017, reflecting demographic shifts within the participant population and potentially broader trends in endurance-sport participation (Nikolaidis and Knechtle, 2019).

### Section 2

In this section, we summarize the physiological findings reported in studies of the *Comrades Marathon*, including responses to heat stress, dysnatremia and exercise-associated hyponatremia (EAH), cardiovascular and locomotor adaptations, skeletal-muscle damage, immune perturbations, nutritional considerations, gastrointestinal disturbances, and broader metabolic consequences.

### Heat stress

The *Comrades Marathon* is traditionally held in late May or early June, a period generally associated with relatively low environmental heat stress. In 2022, however, the race was staged in August, prompting several investigations into the health implications of the altered climatic conditions (Henst et al., 2015; Havenga et al., 2022; Havenga et al., 2024). Across studies, runners were typically exposed to moderate-risk heat stress conditions during the event (Henst et al., 2015; Havenga et al., 2024). Modelling work further indicated that the lowest heat-stress risk along the route occurs between mid-June and mid-July, whereas shifting the race to August substantially increases the likelihood of exposure to ‘strong’ and ‘very strong’ heat-stress periods compared with the traditional race dates (Henst et al., 2015; Havenga et al., 2022; Havenga et al., 2024).

One study deployed seven weather stations, seven PM_2_._5_ monitors, and a spore trap along the 90 km course to characterize microclimatic variability, allergenic aerospora, and particulate-matter exposure during the August race. These measurements confirmed moderate-risk heat-stress conditions and additionally revealed elevated—and potentially harmful—PM_2_._5_ concentrations in spectator-dense areas, likely linked to small fire events associated with race-day festivities (Havenga et al., 2024).

### Dysnatremia and exercise-associated hyponatremia

Runners in the *Comrades Marathon* appear to be at measurable risk for disorders of fluid and electrolyte balance (Hew-Butler et al., 2012). Exercise-associated hyponatremia (EAH) should be considered in any athlete presenting to the medical area with vomiting, altered mental status, and a history of high fluid intake (Hew-Butler et al., 2012). In one study of medical encounters (MEs), fluid and electrolyte disturbances occurred at a prevalence of 8.8 per 1,000 race entrants (Sewry et al., 2022). Both EAH (Noakes et al., 1990; Hew-Butler et al., 2007) and hypernatremia (Hew-Butler et al., 2007; Hew-Butler et al., 2008) have been documented among finishers.

The prevalence of EAH in the *Comrades Marathon* is relatively low, generally below 2% (Noakes et al., 1990; Hew-Butler et al., 2007). Symptomatic hyponatremia is rare, occurring in fewer than 0.3% of competitors during prolonged exercise, even when sodium intake is minimal. However, it is disproportionately represented among collapsed runners, affecting ∼9% of those presenting with collapse (Noakes et al., 1990).

In contrast, hypernatremia appears to be substantially more common. In the 2005 race, 45% of collapsed runners were hypernatremic (Hew-Butler et al., 2007), and in the 2006 edition, hypernatremia was present in 58% of all collapsed runners (Hew-Butler et al., 2008). Both hypo- and hypernatremic athletes require medical management with intravenous fluid therapy (Hew-Butler et al., 2008; Hew-Butler et al., 2012). For EAH, correction of serum sodium does not occur with intravenous Ringer’s lactate but does improve with isotonic or hypertonic saline administration (Hew-Butler et al., 2012). In hypernatremic runners, intravenous infusion of 1 L of either hypotonic or isotonic fluid produced no adverse biochemical or cardiovascular effects and is considered a safe and effective treatment strategy for collapsed athletes in this context (Hew-Butler et al., 2008).

### Effects on the cardiovascular system

Five studies have examined the impact of the *Comrades Marathon* on the cardiovascular system (Noakes et al., 1977; Olivier et al., 1978; George et al., 2009; Mann et al., 2015; Brill et al., 2023). Pre-race screening data derived from two voluntary, open-ended questions indicated that 30% of entrants reported at least one cardiovascular disease (CVD) risk factor, with age >45 years and male sex (27.5%) being the most common (Brill et al., 2023). These findings highlight that a substantial proportion of participants begin the race with identifiable cardiovascular risk profiles.

Cardiac investigations in the *Comrades Marathon* date back to the 1970s. A 1977 report documented six cases of myocardial infarction among participants, including one fatality (Noakes et al., 1977). In 1978, 21 of 48 runners exhibited pre-race electrocardiographic abnormalities suggestive of early ischemic heart disease (Olivier et al., 1978). Collectively, these early studies demonstrated that ultra-marathon runners are not immune to coronary artery disease or atherosclerosis, and that high aerobic fitness does not preclude the presence of clinically significant cardiovascular pathology (Noakes et al., 1977).

More recent work has focused on biochemical and functional cardiac responses to the event. Two studies reported increases in cardiac biomarkers following the race. Total serum creatine kinase (CK) activity rises substantially after the *Comrades Marathon* (Olivier et al., 1978), and elevations in cardiac troponin T (cTnT) have also been observed (Mann et al., 2015). Among runners with increased CK, 50% demonstrated a detectable CK-MB (Creatine Kinase-Myocardial Band) fraction post-race, although this fraction is considered an unreliable marker of myocardial injury following strenuous endurance exercise (Olivier et al., 1978).

Echocardiographic assessments further indicate transient functional cardiac changes associated with participation. Running the *Comrades Marathon* reduces left ventricular ejection fraction (from 71 ± 5% to 64 ± 6%) and decreases the ratio of early to atrial (E/A) peak transmitral flow velocities (from 1.47 ± 0.35 to 1.25 ± 0.30) (George et al., 2009). These alterations are consistent with exercise-induced cardiac fatigue, a well-described but typically reversible phenomenon observed in prolonged endurance events.

### Effects on the locomotor system and skeletal muscles

Running-related injuries (RRIs) are common among ultra-marathoners, and several studies have examined their occurrence in the context of the *Comrades Marathon*. One investigation into gradual-onset running-related injuries (GORRIs) identified a history of chronic disease, a history of allergies, fewer weekly training sessions, and more years of recreational running experience as significant risk factors (Slabber et al., 2023).

Only a limited number of studies have explored the direct effects of the *Comrades Marathon* on the locomotor system (Peters et al., 2004; Hagemann et al., 2009). Participation in the race induces post-race muscle inflammation (Peters et al., 2004). MRI-based assessments revealed no evidence of bone bruising, cartilage defects, or meniscal abnormalities in the knees of participants (Hagemann et al., 2009). However, runners who began the race with pre-existing tendinopathy experienced a worsening of their condition following the event (Hagemann et al., 2009).

A study examining pre-race training and overuse injuries among runners preparing for the 2018 *Comrades Marathon* reported an RRI incidence of 8 injuries per 1,000 hours of running, with an overall injury proportion of 40% (Burgess et al., 2025). Muscles were the most frequently injured structures (47%), followed by tendons (24%). The knee (26%) and hip (19%) were the most commonly affected anatomical regions. Lower training-load distance in the 12 weeks preceding the race was associated with a higher risk of injury—particularly injuries occurring during or after the race—and was a stronger predictor of injury than age or sex. Weekly training frequency demonstrated a heterogeneous relationship with injury risk, and the acute-to-chronic workload ratio showed minimal influence (Burgess et al., 2025).

### The immune system in the *Comrades Marathon*

Several studies have examined the effects of the *Comrades Marathon* on immune function (Nieman et al., 2000; Peters et al., 2001; Peters et al., 2004; Peters et al., 2004). Faster and better-trained runners demonstrated a higher incidence of post-race upper respiratory tract infection (URTI) (55.6%) compared with slower and less-trained runners (40%) (Peters et al., 2004). Race time also appeared to influence post-race inflammatory responses: although faster runners showed no evidence of delayed recovery in total leukocyte, neutrophil, or total and differential lymphocyte counts, slower and less-trained runners exhibited markedly higher C-reactive protein (CRP) concentrations (Peters et al., 2004). The incidence of URTI peaked during the four weeks preceding the race and again between seven and fourteen days post-race, with ∼60% of runners reporting pre-race URTI symptoms also reporting symptoms during the 7–14-day post-race period (Peters et al., 2010).

The potential role of vitamin C supplementation in modulating immune responses has also been investigated. Post-race vitamin C intake was associated with lower serum cortisol, plasma adrenaline, IL-10, and IL-1Ra concentrations (Peters et al., 2001), while daily supplementation with 1,500 mg of vitamin C for one week attenuated the post-race increases in IL-6, IL-10, IL-1Ra, and IL-8 (Nieman et al., 2000).

Another important immunological aspect is endotoxemia in exhausted runners. Athletes completing the race in under eight hours had significantly lower average plasma endotoxin concentrations than those finishing in more than eight hours (Brock-Utne et al., 1988). Gastrointestinal symptoms were strongly associated with endotoxemia: 80.6% of runners with high plasma endotoxin values reported nausea, vomiting, and/or diarrhea, compared with 17.7% of those with low values (Brock-Utne et al., 1988). Elevated endotoxin concentrations typically returned to normal within one to three weeks after the race, during which most runners demonstrated increased anti-endotoxin IgG concentrations (Brock-Utne et al., 1988).

### Nutritional aspects before and during the *Comrades Marathon*

Nutrition is a key component of ultra-marathon performance, yet only two studies have examined this aspect in the context of the *Comrades Marathon*. One study assessed energy intake and supplement use before the race and found no significant association between energy or micronutrient intake and race performance (Peters and Goetzsche, 1997). The relative contribution of carbohydrate to total energy intake increased from 50.0% and 49.5% during training to 57.7% and 56.4% in the pre-race diets of male and female finishers, respectively (Peters and Goetzsche, 1997). A study investigating cases of acute renal failure (ARF) reported that affected runners had consumed an anti-cramp electrolyte supplement; however, the average supplemental intake of sodium (452 mg), potassium (393 mg), calcium (330 mg), and magnesium (154 mg) before and during the race did not exceed recommended upper daily limits (Boulter et al., 2011).

The female athlete triad (FAT) has also been explored in the *Comrades Marathon*. One-third of female participants demonstrated disordered eating behaviors, with nearly half reporting restrictive eating patterns (Folscher et al., 2015). Awareness of the FAT was low, with only 7.5% of female runners familiar with the condition despite 44.1% being classified as high risk (Folscher et al., 2015). Supplement use was common: 78% of female and 62% of male runners reported using vitamin and mineral supplements in their training diets (Peters and Goetzsche, 1997).

### The gastrointestinal system in the *Comrades Marathon*

Runners competing in the *Comrades Marathon* appear to be at measurable risk for gastrointestinal (GI) disturbances. A study investigating the prevalence of medical encounters (MEs) reported a GI-related ME rate of 2.9 per 1,000 race entrants (Sewry et al., 2022). Evidence regarding hepatic responses to the race is limited. One case report described marked elevations in alanine aminotransferase (ALT; 159 U/L, normal range 1–25 U/L) and gamma-glutamyl transferase (GGT; 175 U/L, normal range 0–65 U/L), potentially attributable to the ingestion of painkillers (six tablets containing paracetamol 500 mg/chlormezanone 100 mg) before and during the race (van Zyl-Smit et al., 2000).

### Metabolic aspects in the *Comrades Marathon*

The first studies examining metabolic responses to the *Comrades Marathon* were conducted in the 1980s (McKechnie et al., 1982). In runners completing the race, post-race concentrations of serum sodium, glucose, free fatty acids, glycerol, lactate, pyruvate, and growth hormone were all significantly elevated, whereas serum insulin levels were reduced and serum potassium and triglyceride concentrations remained unchanged (McKechnie et al., 1982).

### Section 3

In this section, we summarize the evidence on medical encounters (MEs) reported during the *Comrades Marathon*, including the prevalence and characteristics of muscle cramping, acute kidney injury and acute renal failure, and post-race recovery dynamics. We also highlight documented differences between Up Runs and Down Runs, particularly regarding patterns of muscle damage and associated clinical presentations.

### Medical encounters in the *Comrades Marathon*

Medical encounters (MEs) during the *Comrades Marathon* have been examined across five studies (Sewry et al., 2022; Brill et al., 2023; Leppan et al., 2023; Sewry et al., 2024; Green et al., 2025). Reported prevalence rates varied considerably, ranging from ∼8.7 per 1000 race starters (Green et al., 2025) to 19–20 per 1000 starters in other cohorts (Sewry et al., 2022; Brill et al., 2023). Serious or life-threatening MEs occurred at a rate of about 1.8 per 1000 entrants (range 1.6–2.1) (Sewry et al., 2022). Entrants reporting allergies, chronic medical conditions, or prescription medication use demonstrated significantly higher ME rates (Brill et al., 2023).

Across organ systems, the most frequently affected categories were fluid and electrolyte disturbances (8.8 per 1000 starters; 8.3–9.4), central nervous system complications (4.0 per 1000; 3.7–4.5), and gastrointestinal issues (2.9 per 1000; 2.6–3.2). Dehydration (7.5 per 1000; 7.0–8.1) and exercise-associated muscle cramping (3.2 per 1000; 2.9–3.6) were the two most common specific diagnoses (Sewry et al., 2022). Another study identified cardiovascular conditions as the largest contributor to MEs, with an incidence of 0.4 per 1000 starters; acute coronary syndrome (ACS) was the most frequent cardiovascular diagnosis (0.2 per 1000) (Green et al., 2025). Notably, no sudden cardiac deaths or cardiac arrests were reported in this dataset (Green et al., 2025).

A large cohort study of 103,131 starters (2014–2019) identified both intrinsic (female sex, faster or slower race pace) and extrinsic (higher wet-bulb globe temperature, Down Run) risk factors for MEs (Sewry et al., 2024). Pre-race medical screening data from 133,641 entrants in the same period showed that 6.9% reported chronic medical conditions and/or prescription medication use, while 7.4% reported allergies (Brill et al., 2023).

Two studies have evaluated the effectiveness of pre-race medical screening instruments. One relied on two voluntary, open-ended questions (Brill et al., 2023), but the study did not describe the context in which these questions were administered nor report any findings regarding their diagnostic yield or effectiveness. As a result, the utility of this minimal screener for identifying at-risk participants remains unclear. In contrast, another study compared short versus comprehensive questionnaires (Leppan et al., 2023) and demonstrated that abbreviated screening tools substantially underestimate chronic medical conditions, allergies, and the proportion of entrants at elevated risk for MEs relative to a full instrument (Leppan et al., 2023). Taken together, these findings support the use of comprehensive pre-race medical screening tools to more accurately identify participants at increased medical risk.

Collectively, the evidence underscores the need for preventive strategies that identify risk factors, guide risk management, and enhance preparedness among athletes, race organizers, and medical teams to reduce the burden of MEs during the event (Havenga et al., 2024).

### The aspect of muscle cramping

The prevalence of a history of exercise-associated muscle cramping (EAMC) among ultra-marathon runners is high, and athletes competing in the *Comrades Marathon* appear to have a particularly elevated risk. In one study investigating medical encounters (MEs), the prevalence of EAMC-related MEs was 3.2 per 1,000 race entrants (Sewry et al., 2022). Another study identified several independent risk factors for EAMC in this event, including a one-unit increase in a composite disease score, a history of collapse, previous chronic musculoskeletal (MSK) injury, and MSK injury within the preceding 12 months (Macmillan et al., 2024). Entrants presenting with these characteristics demonstrated the highest prevalence of EAMC (Macmillan et al., 2024).

Training-related risk factors were also evident. An increase of 10 km in weekly running distance was associated with elevated EAMC risk, as was slower training pace, with each 1 min·km^-^¹ reduction in pace linked to increased likelihood of cramping (Macmillan et al., 2024). These findings suggest that both intrinsic health-related factors and modifiable training characteristics contribute meaningfully to EAMC susceptibility in *Comrades Marathon* participants.

### Acute kidney injury and acute renal failure

Acute kidney injury (AKI) and acute renal failure (ARF) have been recurring research themes in the *Comrades Marathon* since the 1970s, with evidence drawn from case reports (MacSearraigh et al., 1979; Irving et al., 1990; van Zyl-Smit et al., 2000) and field studies (Seedat et al., 1989-1990; Boulter et al., 2011).

The incidence of ARF has been estimated at 1 per 4,125 runners (Boulter et al., 2011). The development of ARF during the race is multifactorial, typically arising from the combined effects of rhabdomyolysis, dehydration, hypotension, hyponatremia, nonsteroidal anti-inflammatory drug (NSAID) use, and hyperuricemia (Seedat et al., 1989-1990; Boulter et al., 2011). Early case series from the 1970s reported 10 cases of ARF over a nine-year period, of which three required hemodialysis and one required peritoneal dialysis (MacSearraigh et al., 1979).

Subsequent field investigations demonstrated that runners who developed ARF commonly presented with rhabdomyolysis, hyponatremia, NSAID use, consumption of anti-cramp electrolyte supplements, and were often novice participants (Boulter et al., 2011). NSAID use during the race appears to be a particularly important contributor (Seedat et al., 1989-1990; van Zyl-Smit et al., 2000). In one case report, a runner ingested six tablets containing paracetamol 500 mg/chlormezanone 100 mg for anticipated musculoskeletal pain and subsequently developed acute tubular necrosis (van Zyl-Smit et al., 2000). However, renal dysfunction may also occur in the context of excessive fluid restriction during the race (Irving et al., 1990).

### Recovery aspects after the race

One study investigated the time course of recovery of muscle function and heart-rate responses following the *Comrades Marathon*. Vertical jump height was significantly reduced immediately after the race, and squat-jump height remained impaired for up to 18 days. The reduction in squat-jump height was substantial, with decreases of up to 20 cm immediately post-race. Leg-extensor muscle power, reflected by vertical-jump performance, showed a similarly prolonged impairment lasting up to 18 days. In addition, runners demonstrated a delayed and exaggerated heart-rate response during steady-state running at moderate to high intensities for as long as 25 days after the race (Chambers et al., 1998).

### Differences between Up Runs and Down Runs

The Down Run is consistently associated with higher markers of skeletal-muscle damage compared with the Up Run (Milledge, 1977). Early work was among the first to demonstrate that the Down Run elicited significantly greater elevations in creatine kinase (CK) than the Up Run, underscoring the substantial eccentric loading imposed by the predominantly downhill course (Milledge, 1977). The alternating race direction therefore provides a unique natural model for examining the differential effects of concentric- versus eccentric-dominated loading on muscle injury. Subsequent studies confirmed these findings, reporting significantly higher post-race CK concentrations after the Down Run compared with the Up Run (Noakes and Carter, 1982).

## Discussion

**Figure 1:**
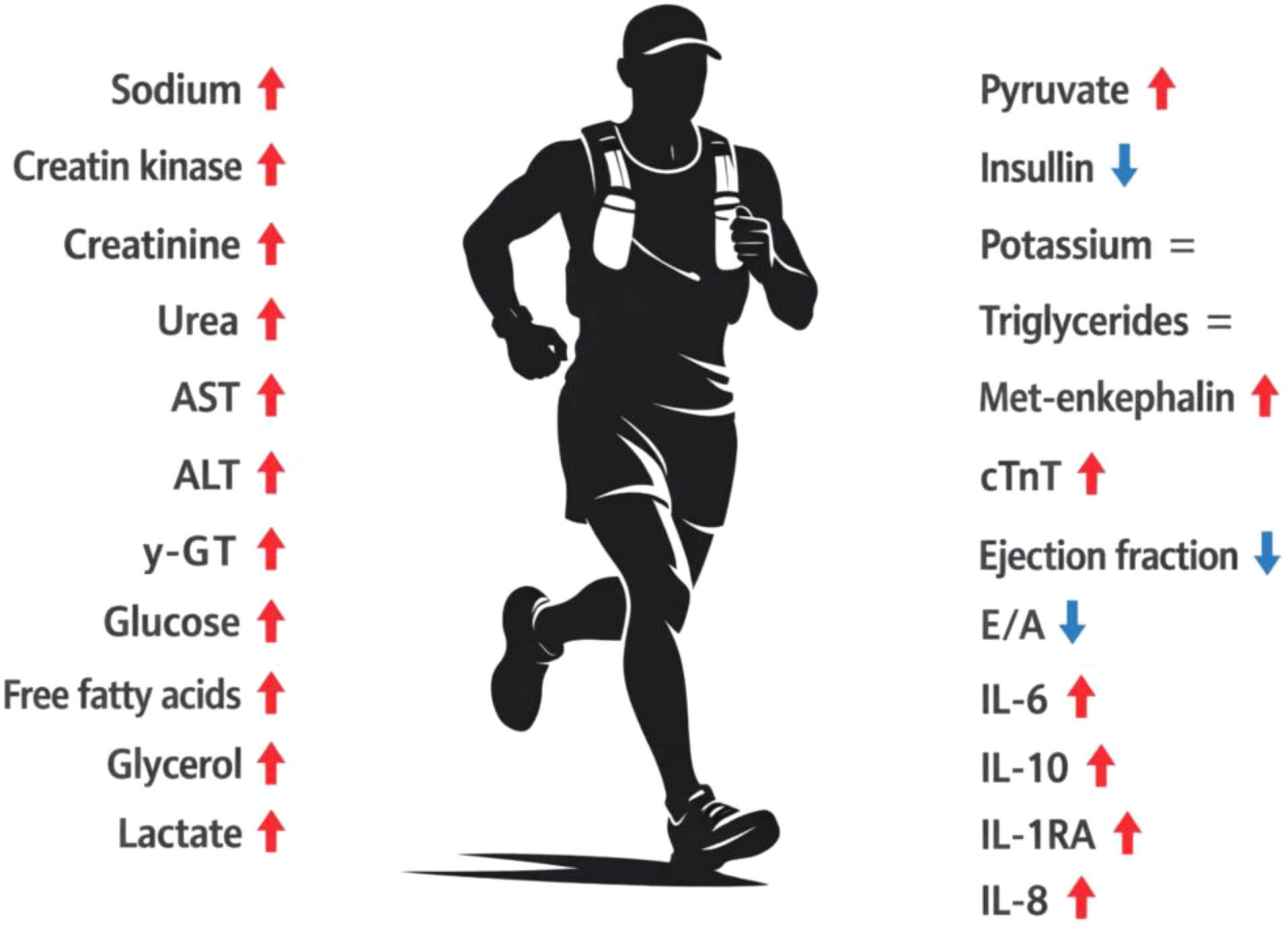
Schematic representation of systemic physiological changes observed in ultramarathon runners competing in the *‘Comrades Marathon’.* Red arrows pointing upwards (↑) indicate a significant increase, blue arrows pointing downwards (↓) indicate a significant decrease, and gray equal signs (=) represent stable parameters.

This review aimed to synthesize the current body of scientific knowledge derived from studies involving participants in the *Comrades Marathon* or reporting on their performance and physiological responses. **Figure 1** provides an overview of all reported changes in blood-based biomarkers across the included studies, summarizing the key biochemical alterations associated with participation in this event.

### Section 1

In this section, we discuss the findings from *Comrades Marathon* research on participation and performance trends, the age of peak performance, and pacing strategies, and we interpret these results in the context of the broader ultra-endurance literature.

### Participation and performance trends

Participation and performance trends in the *Comrades Marathon* reflect both the historical expansion of ultra-endurance running and evolving competitive standards. Although men continue to outperform women across all age groups, the progressive narrowing of sex-based performance differences mirrors patterns observed in other endurance disciplines and likely reflects improvements in training quality, professionalization, and competitive depth among female athletes (Nikolaidis and Knechtle, 2019). Importantly, performance gains appear to be driven primarily by elite runners rather than the broader field, underscoring the race’s value as a model for long-term performance analyzes and elite-level progression (Zingg et al. 2013a; Zingg et al. 2013b).

Studies examining participation and performance trends in the *Comrades Marathon* have consistently shown a low proportion of female entrants (Nikolaidis et al., 2021), a pattern mirrored in other ultra-marathons (Thuany et al., 2022; Thuany et al., 2024). The growth in total finishers over time has been driven primarily by increased participation among age group runners (Nikolaidis et al., 2021), a trend also observed in other time-limited and distance-limited (Knechtle et al., 2019) ultra-endurance events. Across the years, running speed has improved in both women and men (Nikolaidis and Knechtle, 2019; Nikolaidis et al., 2021), and similar performance gains have been documented in other iconic ultra-marathons such as the *Spartathlon* (Knechtle et al., 2021).

Men have consistently outperformed women across all performance levels and age groups in the *Comrades Marathon* (Nikolaidis et al., 2021), reflecting the well-described male dominance in ultra-marathon running (Cejka et al., 2014). However, this sex-based performance gap narrows substantially with advancing age. In 100-km ultra-marathons, it disappears entirely in athletes older than 60 years (Stöhr et al., 2021). Moreover, in a large dataset of more than 1,100,000 race records from Switzerland (1999–2019), female ultra-marathoners aged 75 years and older demonstrated a performance difference of less than 4% compared with their male counterparts across distances ranging from 5 km to the ultra-marathon (Knechtle et al., 2023). Participation in other ultra-marathons such as the *Western States Endurance Run* has increased between 1974 and 2007 among women and older athletes, and the ages of the fastest runners at the *Western States Endurance Run* have gradually risen to the extent that these runners are older than the ages at which the fastest marathons are run. In contrast to what has been observed for men, finish times have improved for the top women across the last two decades at the *Western States Endurance Run* (Hoffman and Wegelin, 2009).

Nationality-related performance differences have also been reported. Runners from Russia were the fastest, whereas those from India were the slowest (Nikolaidis and Knechtle, 2019). The prominence of Russian athletes is noteworthy in light of the historical context. International participation in the *Comrades Marathon* was restricted during the Apartheid era and increased markedly after 1994, reshaping the competitive field (Merrett, 2007).

### The age of peak performance

The age of peak performance in the *Comrades Marathon* occurs later than in shorter endurance events, highlighting the importance of accumulated experience, pacing proficiency, and long-term endurance-specific adaptations. Sex-specific differences in peak age vary by performance level, suggesting distinct developmental trajectories for male and female athletes (Knechtle and Nikolaidis, 2017). Collectively, these findings reinforce that ultra-endurance success depends less on maximal physiological capacity and more on sustained training exposure, strategic execution, and cumulative adaptation over many years.

The best available data indicate that peak performance in the *Comrades Marathon* occurs between 30 and 35 years of age. Large-scale analyzes show that men tend to reach their performance peak slightly earlier (around ∼30 years), whereas women peak later, typically in their mid-30s, resulting in sex-specific rather than a single combined age window (Knechtle and Nikolaidis, 2017; Nikolaidis and Knechtle, 2019). Women often maintain endurance performance longer and may peak later due to differences in muscle metabolism, fatigue resistance, and long-term training trajectories (Devries, 2016). In contrast, men tend to achieve maximal speed-related performance at a younger age (Sitko et al., 2025).

### Pacing during the *Comrades Marathon*

A search of the scientific literature revealed no peer-reviewed studies examining pacing strategies in the *Comrades Marathon*. Aside from an online pacing calculator (Coach Norrie Calculator, [[NoYear]]), no empirical analyzes of split-time dynamics, pacing variability, or sex- and age-related pacing differences have been conducted for this event. This stands in contrast to marathon and ultra-marathon research more broadly, where pacing is recognized as a critical determinant of performance.

In marathon running, pacing strategies have been systematically reviewed and shown to influence performance across sex, age, and competitive level (Sha et al., 2024). In ultra-marathon events, several studies have demonstrated that pacing variability, course profile, and athlete characteristics strongly shape performance outcomes.

In the *Western States Endurance Run*, runners exhibit substantial speed variability between checkpoints in both sexes, with predominantly positive pacing and pronounced effects of elevation changes on speed fluctuations. Men typically start faster and decelerate more than women, and pacing differs across male—but not female—age groups. Notably, the fastest and slowest runners show less pacing variability than mid-level performers (Markovic et al., 2025).

In the *Spartathlon*, successful finishers display a reverse J-shaped pacing pattern, with speed declining from the start to the seventh checkpoint and increasing thereafter, likely reflecting the race’s distinctive elevation profile. Men show larger early-race speed changes, whereas women show greater variation at the final checkpoint. Although age and sex do not influence average checkpoint speed, performance groups differ markedly, with the fastest and slowest runners again demonstrating fewer speed changes than intermediate groups (Knechtle et al., 2022).

In 100-km ultra-marathons, faster athletes maintain more stable pacing profiles, beginning at higher speeds, sustaining them for longer durations, and exhibiting fewer fluctuations than slower runners (Lambert et al., 2004).

Despite the clear importance of pacing in endurance performance, scientific analyzes of pacing in the *Comrades Marathon* remain absent. Several factors may contribute to this gap. The alternating race direction—Up Run versus Down Run—introduces substantial variation in elevation and biomechanical demands, complicating year-to-year comparisons. Additionally, the availability, consistency, and granularity of split-time data may have limited researchers’ ability to conduct detailed checkpoint-based analyzes.

Given the scale, history, and unique course characteristics of the *Comrades Marathon*, the lack of empirical pacing research represents a significant gap in the literature, particularly when contrasted with the extensive pacing analyzes conducted in other major ultra-marathon events.

### Section 2

In this section, we discuss the physiological findings reported in studies of the *Comrades Marathon*, including responses to heat stress, dysnatremia and EAH, cardiovascular and locomotor adaptations, skeletal-muscle damage, immune perturbations, nutritional considerations, gastrointestinal disturbances, and broader metabolic consequences. These topics are interpreted in relation to the existing literature to contextualize the multisystem demands of this ultra-marathon.

### The locomotor system and running-related injuries in the *Comrades Marathon*

Injuries related to preparing for or competing in the *Comrades Marathon* have been examined in several studies (Peters et al., 2004; Hagemann et al., 2009; Slabber et al., 2023; Burgess et al., 2025). Pre-race, the knee and hip were the most commonly affected regions (Burgess et al., 2025). During the race, muscle inflammation was observed (Hagemann et al., 2009), although MRI investigations showed no major structural abnormalities in the knees of participating runners (Hagemann et al., 2009). Similar findings have been reported in other ultra-endurance events. Research from the *TransEurope FootRace* (TEFR) demonstrated no major MRI-detectable knee abnormalities (Schütz et al., 2020) and instead suggested a regenerative process during this multi-stage ultra-marathon (Schütz et al., 2020). No relevant negative effects on periarticular knee tissues were identified (Schütz et al., 2020; Schütz et al., 2014), supporting the notion that knee cartilage in ultra-marathoners may reorganize and adapt to prolonged loading demands (Schütz et al., 2020).

From a biomechanical perspective, Gallant and Pierrynowski (2014) proposed that running-related injuries may arise from high impact forces during the stance phase, which can impair subtalar joint motion. Given the tightly linked kinematic chain of the lower limbs, dysfunction at the subtalar joint may propagate proximally, affecting more central joints and surrounding anatomical structures. In addition, previous injury within the past year is considered the strongest predictor of future running-related injury (Saragiotto et al., 2014). The *Comrades Marathon* Down Run (Pietermaritzburg to Durban), which involves ∼2,000 m of cumulative descent, imposes substantial eccentric loading on the quadriceps and patellofemoral joint, further elevating musculoskeletal stress and injury risk.

### Muscle damage in the *Comrades Marathon*

The *Comrades Marathon* provides a natural experiment for studying muscle damage because the race direction alternates annually. The Down Run consistently produces greater skeletal muscle damage than the Up Run, as shown by markedly higher post-race creatine kinase (CK) concentrations. This difference is driven by the eccentric-dominant loading imposed by prolonged downhill running, making the *Comrades Marathon* an effective model for contrasting eccentric-biased versus concentric-biased muscular stress (Milledge, 1977; Noakes and Carter, 1982).

Muscle damage has also been extensively documented in the *Western States Endurance Run*. In the 2010 edition, CK concentrations were measured in 216 of the 328 finishers (66%). The median CK was 20, 850 IU/L, the mean was 32,956 IU/L, and values ranged from 1,500 to 264,300 IU/L. Thirteen runners (6%) exceeded 100,000 IU/L. These CK values were significantly higher than those reported in the 1995 *Western States Endurance Run*, indicating either increased physiological strain or differences in environmental or methodological factors (Hoffman et al., 2012a). Notably, CK concentration showed no significant association with finish time, age, gender, or running experience, highlighting the large interindividual variability in susceptibility to muscle damage. Blood sodium concentrations were available for a subgroup of 159 runners, but no significant relationship was found between sodium levels and CK, suggesting that electrolyte status does not meaningfully influence the magnitude of muscle damage in this context (Hoffman et al., 2012a).

The role of NSAIDs, particularly ibuprofen, has been evaluated in participants in *Western States Endurance Run*. Ibuprofen use did not reduce muscle damage or soreness, and was instead associated with higher levels of endotoxemia and inflammation (Nieman et al., 2006). Further analyzes showed that muscle damage was significantly correlated with post-race delayed-onset muscle soreness (DOMS) and increases in five of seven measured cytokines. NSAID users did not experience any reduction in muscle damage or DOMS, but they did exhibit higher post-race plasma concentrations of five cytokines, indicating an amplified inflammatory response (Nieman et al., 2005).

Across both *Comrades* and *Western States Endurance Run*, the evidence converges on a clear pattern. Extreme eccentric loading, not race duration, pace, demographics, sodium status, or NSAID use, is the primary driver of skeletal muscle damage, and NSAIDs may worsen systemic inflammation without reducing muscle injury or soreness.

### Electrolyte metabolism during the *Comrades Marathon*

In this subsection, we examine electrolyte-related disturbances reported in the Comrades Marathon, focusing on three key areas: hyponatremia, collapsed runners, and hypernatremia. These categories reflect the primary electrolyte abnormalities documented in the literature and provide a framework for interpreting the diverse clinical presentations observed during this event.

#### Hyponatremia

EAH is a recognized complication of prolonged endurance exercise, yet its prevalence varies markedly across events. In the *Comrades Marathon*, EAH remains relatively uncommon among finishers, with consistently low rates reported across multiple cohorts (Noakes et al., 1990; Hew-Butler et al., 2007). In contrast, the *Spartathlon* demonstrates one of the highest documented EAH prevalences in ultra-endurance sport. Among 63 successful finishers, 65% developed hyponatremia, with 43% presenting with mild and 22% with severe EAH (Seal et al., 2019). This exceptionally high burden is plausibly attributable to the race’s extreme duration, substantial elevation change, and often challenging environmental conditions (Seal et al., 2019).

Comparable findings have been reported in other 100-mile events. In the 2009 *Western States Endurance Run*, 30% of monitored participants met biochemical criteria for EAH (post-race [Na^+^] <135 mmol/L) (Hoffman et al., 2012b). Notably, post-race [Na^+^] was directly related to percentage body mass change. However, half of the athletes with EAH exhibited body mass losses of 3–6%, demonstrating that EAH can occur despite net dehydration (Hoffman et al., 2012b). EAH in *Western States Endurance Run* was unrelated to age, sex, finish time, or NSAID use, although affected athletes had completed fewer prior 161-km ultramarathons, suggesting a potential protective effect of experience (Hoffman et al., 2012b). Creatine kinase concentrations showed no significant association with post-race [Na^+^] (Hoffman et al., 2012b).

Severe clinical consequences have also been documented. More than 1% of participants in the *Western States Endurance Run* required hospitalization for hyponatremia combined with rhabdomyolysis, and a subset progressed to acute renal failure (Bruso et al., 2010). Those affected tended to be younger, faster, more likely to have experienced a training-limiting injury, and more likely to have used NSAIDs during the race (Bruso et al., 2010). Elevated initial blood urea nitrogen and creatinine, but not creatine kinase, differentiated those who developed acute renal failure (Bruso et al., 2010).

Importantly, sodium intake appears to play only a minor role in the development of EAH during continuous exercise lasting up to 30 hours. Evidence from *Western States Endurance Run* indicates that overhydration is the primary driver, whereas low sodium intake in supplements contributes minimally (Hoffman and Stuempfle, 2015). Consequently, avoiding excessive fluid intake remains the most effective preventive strategy for EAH in long-duration ultramarathons (Hoffman and Stuempfle, 2015).

Within this context, the extreme EAH prevalence in *Spartathlon* underscores the need for targeted athlete education, careful hydration strategies, and on-course medical vigilance.

#### Hypernatremia

The prevalence of hypernatremia in the *Comrades Marathon* exceeds that of EAH (Noakes et al., 1990; George et al., 2009), a pattern partly shaped by case-ascertainment bias because many hypernatremic cases are detected among collapsed runners undergoing medical evaluation (Krabak et al., 2017; Schwellnus et al., 2019a; Adrogué et al., 2022; Altieri et al., 2026). Despite this higher frequency, the clinical severity profiles diverge markedly. Hypernatremia in ultra-endurance events is typically mild, dehydration-related, and rapidly reversible with oral or intravenous rehydration (Krabak et al., 2017). In contrast, even though EAH is less common, its pathophysiological mechanism—water retention and dilutional hypo-osmolality—can precipitate cerebral edema, making symptomatic or encephalopathic EAH the more acutely dangerous dysnatremia (Altieri et al., 2026). Thus, incidence patterns alone can be misleading. Hypernatremia is more prevalent, but EAH carries the substantially greater risk of life-threatening neurological compromise.

#### Collapsed runners

Population-based studies consistently demonstrate that the prevalence of EAH in the *Comrades Marathon* is low, generally <2% among unselected finishers (Noakes et al., 1990; Hew-Butler et al., 2007). Symptomatic EAH is even rarer, occurring in <0.3% of competitors during prolonged exercise, even when sodium intake is minimal. In contrast, EAH is disproportionately represented among collapsed runners, affecting ∼9% of those presenting with collapse (Noakes et al., 1990).

This discrepancy reflects a fundamental methodological issue. Medical-tent cohorts are clinically filtered and therefore subject to strong selection bias. Medical-tent data capture only athletes whose symptoms are severe enough to prompt self-presentation or require evacuation (Schwellnus et al., 2019a). As a result, these cohorts are non-random and symptom-enriched, overrepresenting conditions that predispose to collapse—such as EAH, heat illness, or exertional collapse—relative to their true prevalence in the full race population. By contrast, population-based sampling of unselected finishers provides an unbiased estimate of epidemiological burden and consistently demonstrates low EAH prevalence. Thus, medical-tent prevalence reflects clinical burden among those requiring care, whereas finisher-based prevalence reflects true population prevalence.

Failure to account for this recruitment bias risks misinterpretation of risk, overestimation of rare conditions, and inappropriate extrapolation of medical-tent findings to the broader athlete population. Studies should therefore clearly differentiate between population prevalence and clinical prevalence, explicitly define the sampling frame, and avoid generalizing from medically selected cohorts to all race participants.

A potential criticism is that the higher proportion of hyponatremic athletes observed in medical-tent cohorts contradicts the low population prevalence. However, this divergence is expected and methodologically coherent. Medical-tent cohorts inherently oversample symptomatic individuals and therefore cannot be used to infer population-level risk. Treating these clinically filtered samples as epidemiological denominators would inflate the apparent prevalence of EAH and distort hydration-related risk communication. Our interpretation explicitly distinguishes these sampling frames, ensuring that clinical prevalence is not misrepresented as population prevalence.

Importantly, dysnatremia among collapsed runners is not limited to hyponatremia. Hypernatremia appears substantially more common. In the 2005 *Comrades Marathon*, 45% of collapsed runners were hypernatremic (Hew-Butler et al., 2007), and in the 2006 *Comrades Marathon*, hypernatremia was present in 58% of all collapsed runners (Hew-Butler et al., 2008). These findings indicate that, within the subset of athletes who collapse, dehydration-driven electrolyte imbalance is more prevalent than fluid-overload–associated hyponatremia.

Both hypo- and hypernatremic athletes require medical management with intravenous fluids (Hew-Butler et al., 2008; Hew-Butler et al., 2012). In EAH, serum sodium concentration does not improve with intravenous Ringer’s lactate, whereas isotonic or hypertonic saline effectively corrects hyponatremia (Hew-Butler et al., 2012). Ringer’s lactate is contraindicated in EAH because it is slightly hypotonic relative to the 3% hypertonic saline required for appropriate treatment (Adrogué et al., 2022). In hypernatremic runners, infusion of 1 L of either hypotonic or isotonic fluid produced no adverse biochemical or cardiovascular effects and is considered a safe and effective treatment strategy in this context (Hew-Butler et al., 2008).

Although the overall prevalence of EAH in the *Comrades Marathon* is low, medical-tent data show that a substantial proportion of collapsed runners present with hypernatremia, indicating dehydration-driven electrolyte imbalance rather than fluid overload (Hew-Butler et al., 2007). This pattern underscores the need for individualized hydration strategies and evidence-based on-course medical management to reduce dysnatremia risk and support optimal athlete performance.

### The cardiovascular system in the *Comrades Marathon*

The cardiovascular response to the *Comrades Marathon* is characterized by transient functional and biochemical alterations that typically resolve within hours to days after the race. Although early investigations reported ischemic events in some participants, more recent evidence suggests that enhanced pre-race screening and improved race-day medical preparedness have substantially reduced the incidence of severe cardiovascular complications (Noakes et al., 1977; George et al., 2009).

Historical reports document several deaths during the *Comrades Marathon* (Noakes et al., 1977), and in some cases, pre-race electrocardiographic abnormalities were consistent with early ischemic heart disease (Olivier et al., 1978). However, no formal prevalence data exist for this event. Broader epidemiological analyzes indicate that the risk of death during marathon running is ∼0.67 per 100,000 finishers (Dayer and Green, 2019), and cardiovascular-related deaths in ultra-marathon events appear to be exceedingly rare (Scheer and Rojas-Valverde, 2021). Overall mortality during ultra-marathon running is extremely uncommon (Michigan woman dies during colorado’s hardrock 100 ultramarathon). Notably, the last documented death in the *Comrades Marathon* occurred in the 1970s, suggesting that contemporary pre-race screening protocols and medical oversight have been effective (Paris de Boam et al., 2025).

Increases in cardiac biomarkers such as cardiac troponin T (cTnT) (George et al., 2009) and creatine kinase (CK) (Olivier et al., 1978) during the *Comrades Marathon* are consistent with findings from other ultra-endurance events (Żebrowska et al., 2019; Liu et al., 2021; Burger et al., 2024). Interestingly, no study has examined changes in N-terminal pro–brain natriuretic peptide (NT-proBNP) in this race, despite evidence that NT-proBNP also rises during ultra-marathon running (Liu et al., 2021; Burger et al., 2024).

Interpretation of CK-MB requires particular caution. Although CK-MB was historically used as a marker of myocardial injury, it is now recognized as a poor indicator of cardiac damage in ultra-endurance athletes because it can also be released from skeletal muscle. George et al (George et al., 2009). and the Noakes research group have emphasized that the extreme skeletal-muscle damage incurred during a 90-km race—especially the Down Run—causes substantial elevations in total CK, with proportional increases in CK-MB that may falsely suggest myocardial injury. Earlier studies (Noakes et al., 1977; Olivier et al., 1978) interpreted CK-MB elevations as evidence of ischemia, but current consensus holds that cTnT is the only reliable biomarker for cardiac-specific injury in this context (Tuominen et al., 2025). Consequently, the ischemic changes reported by Olivier et al (Olivier et al., 1978). should be interpreted with caution. George et al (George et al., 2009). demonstrated via speckle-tracking echocardiography that elevated troponins and a modest reduction in left-ventricular ejection fraction (from 71% to 64%) reflect transient “cardiac fatigue” rather than permanent ischemic injury. Their segmental analyzes showed global, not regional, functional reductions—supporting a physiological, reversible response rather than localized myocardial damage.

Electrocardiographic (Olivier et al., 1978) and echocardiographic (George et al., 2009) changes observed in Comrades Marathon participants are consistent with patterns documented in endurance and ultra-endurance athletes more broadly (Wolff et al., 2022). Similar findings have been reported in other prolonged events. For example, completion of the *Western States Endurance Run* produced significant right-sided ECG alterations, with some individuals exhibiting more pronounced responses. Post-race changes in P-wave morphology, ST-segment deviation, and T-wave amplitude are characteristic of acute exercise-induced right-heart electrical adaptation, reflecting transient loading of the right atrium and ventricle during sustained high-volume endurance exercise (Lord et al., 2016). These adaptations appear to be part of the normal physiological response to prolonged exertion rather than markers of pathological myocardial injury.

Prolonged strenuous exercise induces molecular and cellular adaptations, including altered gene expression and remodeling of myocardial proteins (Janik et al., 2025). Importantly, repeated participation in endurance events does not appear to cause long-term deterioration of right-ventricular function in most recreational male athletes (Schindler et al., 2025). Fitness level may modulate these responses: long-term high-intensity endurance exercise has been associated with myocardial stress in amateur runners, whereas highly trained athletes demonstrate more robust myocardial repair capacity (Schindler et al., 2025; Hu et al., 2023). Nevertheless, both biomarker elevations and echocardiographic changes observed during the *Comrades Marathon* are reversible, underscoring their functional and transient nature.

Debate persists over whether endurance exercise causes myocardial injury, as prolonged events often trigger transient rises in troponins and NT-proBNP, biomarkers typically elevated in cardiac disease. Such increases are common in both elite and recreational athletes, yet it remains unclear whether they indicate true cardiac insult or a normal physiological response. These post-exercise elevations can also complicate clinical assessment (Scharhag et al., 2008).

### Effects of the *Comrades Marathon* on the immune system

In the *Comrades Marathon*, faster and better-trained runners have been shown to experience a higher incidence of post-race upper respiratory tract infection (URTI) compared with slower and less-trained participants (Peters et al., 2004). An elevated risk of URTI following marathon and ultramarathon running is well established (Sardeli et al., 2024). This pattern is commonly interpreted through the J-shaped model, which proposes that while moderate-intensity exercise enhances immune competence, prolonged high-intensity exercise may transiently suppress immune function (Gomes and Florida-James, 2016).

Following the *Comrades Marathon*, this transient “open window” of immune suppression—lasting approximately 3 to 72 hours—creates a period of heightened vulnerability. During this time, exposure to high-density spectator environments and international travel, particularly long-haul flights, increases contact with novel pathogens precisely when host defenses are temporarily impaired.

Evidence also suggests that vitamin C supplementation may modulate post-exercise immune and neuroendocrine responses. In the *Comrades Marathon*, post-race vitamin C intake was associated with lower serum cortisol and plasma adrenaline—markers with established physiological relevance—alongside reductions in IL-10 and IL-1Ra, which currently lack direct clinical translation and remain exploratory (Peters et al., 2001). Similarly, daily supplementation with 1,500 mg of vitamin C for one week attenuated post-race increases in IL-6, IL-10, IL-1Ra, and IL-8. These cytokines are widely used as research indicators of inflammatory signaling but do not yet have validated clinical thresholds or diagnostic utility (Nieman et al., 2000).

Comparative data from other ultramarathon events support the complexity of immune responses to prolonged endurance exercise. In the *Western States Endurance Run*, only a modest correlation was observed between cytokines and F(2)-isoprostanes at 90 km—when oxidative stress peaked—and no other significant associations between immune and oxidative stress markers were detected during or after the 160-km race (Nieman et al., 2003). Approximately one in four ultra-marathoners reported URTI symptoms during the two-week post-race period, and a low mid-race salivary IgA secretion rate was the strongest predictor of subsequent URTI occurrence (Nieman et al., 2003), reinforcing the role of mucosal immunity in post-race susceptibility.

Collectively, these findings indicate that the *Comrades Marathon* induces a transient, multifaceted immune perturbation, shaped by exercise intensity, neuroendocrine stress, mucosal immune function, and environmental exposures. While cytokine responses provide valuable mechanistic insight, clinically meaningful predictors of post-race illness—such as sIgA suppression—remain more robust indicators of URTI risk. This highlights the need for individualized recovery strategies and targeted illness-prevention measures during the vulnerable post-race period.

### Effects of the *Comrades Marathon* on the gastrointestinal system

Despite the high prevalence of gastrointestinal (GI) complaints among *Comrades Marathon* participants, the physiological consequences of this event remain insufficiently characterized. The limited evidence available—primarily a single case report describing post-race elevations in liver enzymes—suggests that the GI tract and hepatobiliary system may be vulnerable to the combined effects of prolonged running, heat exposure, and nutritional stress. In the *Western States Endurance Run*, among race finishers, 43.9% reported that GI symptoms affected their race performance, with nausea being the most common symptom (86.0%). Among race non-finishers, 35.6% reported that GI symptoms were a reason for dropping out of the race, with nausea being the most common symptom (90.5%). For both finishers and non-finishers, nausea was greatest during the most challenging and hottest part of the race. GI symptoms are very common during ultramarathon running, and in particular, nausea is the most common complaint for finishers and non-finishers (Stuempfle and Hoffman, 2015).

Insights from other endurance and ultra-endurance events (Knechtle and Nikolaidis, 2018; Braschler et al., 2025), particularly the *Western States Endurance Run* and the *Spartathlon*, help contextualize these observations and highlight potential mechanisms relevant to athletes in the *Comrades Marathon*. Marked increases in aspartate aminotransferase (AST) and alanine aminotransferase (ALT) have been documented after the *Spartathlon*, whereas γ-glutamyl transferase (γ-GT) typically remains unchanged. Although the absolute enzyme values reported in *Spartathlon* athletes exceed those described in *Comrades Marathon*, the pattern of hepatocellular stress is consistent with what would be expected under conditions of reduced splanchnic perfusion, increased gut permeability, and substantial skeletal muscle breakdown. These findings suggest that the *Comrades Marathon* likely induces similar, albeit less extreme, hepatic perturbations. The *Spartathlon* data therefore provide a useful physiological reference point, illustrating the upper limits of exercise-induced hepatic strain in ultra-endurance contexts (Knechtle and Nikolaidis, 2018).

Alterations in GI hormonal regulation observed during the *Spartathlon* further underscore the sensitivity of the gastrointestinal system to prolonged exertion. Divergent gastrin responses between finishers and non-finishers—an increase in the former and a marked decrease in the latter—highlight the complex interplay between exercise intensity, pacing, and splanchnic blood flow. Although the *Comrades Marathon* is shorter and typically less physiologically extreme, comparable mechanisms may contribute to the high incidence of nausea, reflux, and feeding intolerance reported by participants. Athletes experiencing greater physiological strain or suboptimal pacing may be particularly susceptible to suppressed gastrin secretion and impaired gastric emptying (Knechtle and Nikolaidis, 2018).

Preventive strategies explored in the *Spartathlon*, such as prophylactic proton pump inhibitor (PPI) administration, also offer insights relevant to athletes in the *Comrades Marathon*. The observed reduction in GI bleeding with pantoprazole suggests that acid-related mucosal vulnerability increases during prolonged endurance exercise. While routine PPI use cannot be recommended for all participants in *Comrades Marathon*, targeted prophylaxis may be appropriate for athletes with a history of GI bleeding, reflux disease, or NSAID use. These findings emphasize the need for individualized risk assessment rather than universal pharmacological intervention (Knechtle and Nikolaidis, 2018).

Collectively, the available evidence indicates that the GI and hepatic responses observed in the Spartathlon are mechanistically relevant to the *Comrades Marathon*, even if the magnitude of disturbance is likely lower. The paucity of *Comrades Marathon*-specific physiological data highlights a clear gap in the literature and underscores the need for systematic investigation into gastrointestinal function, hepatocellular stress, and protective strategies in this unique ultra-endurance event.

### Metabolism

Running the *Comrades Marathon* leads to increases in serum sodium, glucose, free fatty acids, glycerol, lactate, pyruvate, and growth hormone, accompanied by a reduction in serum insulin and no meaningful changes in serum potassium or triglyceride concentrations (McKechnie et al., 1982). These alterations mirror those observed in shorter (42 km) and longer (160 km) endurance events, suggesting that the overall metabolic response to marathon running (Braschler et al., 2025) and ultra-marathon running (Knechtle and Nikolaidis, 2018) is broadly similar despite substantial differences in race duration, terrain, and intensity. This consistency across distances highlights the robustness of the metabolic pathways supporting prolonged endurance exercise and underscores the central role of substrate mobilization, hormonal regulation, and fluid–electrolyte balance in sustaining ultra-endurance performance.

### Nutritional aspects before and during the *Comrades Marathon*

Nutrition research in the *Comrades Marathon* is limited, and available evidence shows no clear association between pre-race energy or micronutrient intake and performance. Runners typically increase carbohydrate intake in the days preceding the race, and although electrolyte supplements were used by some athletes—including those with acute renal failure (ARF)—intakes remained below established upper daily limits. Among female participants, disordered and restrictive eating patterns were common, awareness of the female athlete triad was low, and vitamin/mineral supplement use was widespread in both sexes (Peters and Goetzsche, 1997; Boulter et al., 2011; Folscher et al., 2015). These findings highlight the need for improved nutritional education, particularly regarding energy availability and sex-specific health risks.

Comparative data from the *Western States Endurance Run* provide additional context for fueling strategies in ultra-endurance events. In that race, the average pre-race breakfast contained 70 ± 16 g carbohydrate, 29 ± 20 g protein, and 21 ± 8 g fat. During the event, athletes consumed 1,162 ± 250 g of carbohydrate (7  ± 20 g/h), with minimal fat and protein intake, resulting in a total caloric intake of 5,530 ± 1,673 kcal (333 ± 105 kcal/h). Notably, 93% of calories were derived from commercial sports nutrition products, and athletes also consumed 912 ± 322 mg of caffeine and 6.9 ± 2.4 g of sodium. Despite limited access to professional nutrition support, athletes practiced fueling strategies that maximized carbohydrate availability and aligned closely with contemporary evidence-based recommendations for ultra-endurance performance (Stellingwerff, 2016).

Together, these findings suggest that while *Comrades Marathon* nutrition research remains sparse, data from comparable ultra-marathon events indicate that high carbohydrate availability, structured fueling plans, and adequate sodium intake are central to supporting performance and mitigating physiological stress. The prevalence of restrictive eating patterns and low awareness of female-specific health risks in participants in the *Comrades Marathon* underscores the importance of targeted nutritional guidance and athlete education.

### Section 3

In this section, we discuss findings from the *Comrades Marathon* on MEs, muscle cramping, AKI and ARF, post-race recovery, and differences between Up Runs and Down Runs in muscle-damage profiles. These observations are integrated with evidence from other ultra-marathon events to contextualize the clinical and physiological challenges unique to the Comrades Marathon.

Across the scientific literature, the most extensively studied topics in the *Comrades Marathon* include fluid and electrolyte disturbances [28, 29, 30, 32], kidney injury [8, 44, 46, 48, 49], and medical encounters [13, 16, 18, 29]. Armstrong (Armstrong, 2021) emphasized that disruptions in fluid–electrolyte balance may arise from both dehydration and overhydration, and that nutritional strategies to prevent such imbalances must account for individual athlete characteristics such as body composition, performance level, and environmental conditions (Amawi et al., 2024). Notably, EAH was first described in the *Comrades Marathon* in the mid-1980s by Timothy Noakes (Knechtle et al., 2019), marking a pivotal moment in the understanding of hydration-related risks in ultra-endurance sport. This early work fundamentally reshaped subsequent research on safe fluid-replacement practices and continues to influence contemporary guidelines for athlete hydration and medical management during prolonged endurance events.

### Medical encounters at the *Comrades Marathon*

Research on medical encounters (MEs) is a distinctive feature of the *Comrades Marathon* literature, with far more detailed reporting than in most other ultra-endurance events (Sewry et al., 2022; Brill et al., 2023; Leppan et al., 2023; Sewry et al., 2024). Only a limited number of comparable investigations exist, including studies from the *Boston Marathon* (Whitney et al., 2025) and the *Two Oceans Marathon* (Schwellnus et al., 2019b). Expanding ME surveillance to other large-scale running events—particularly major city marathons with tens of thousands of participants—would provide valuable comparative data and help contextualize the medical demands of ultra-endurance racing.

Consistent with available evidence, MEs are relatively frequent during the *Comrades Marathon*, with fluid and electrolyte disturbances, central nervous system symptoms, and gastrointestinal complaints representing the most common presentations. The overall incidence of MEs is shaped by a combination of intrinsic factors, such as sex and pacing strategy, and extrinsic influences, including environmental conditions and race direction (Sewry et al., 2022).

These findings highlight the complex interplay between athlete characteristics, race-specific demands, and environmental stressors in determining medical risk during ultra-endurance events.

### Heat stress during the *Comrades Marathon*

The *Comrades Marathon* is held in late autumn to early winter in the Southern Hemisphere, a period during which regional ambient temperatures typically reach ~30 °C and population-level heat-stress risk is generally considered low (Henst et al., 2015; Havenga et al., 2024). However, the race route traverses the Valley of a Thousand Hills, a region characterized by marked microclimatic variability, including stagnant air pockets, reduced convective airflow, and fluctuating particulate matter concentrations (The comrades marathon). These local features can elevate the effective thermal load experienced by runners beyond what would be predicted from ambient temperature alone. Consistent with this, on-course environmental monitoring has shown that athletes are frequently exposed to moderate heat-stress conditions, with wet-bulb globe temperature (WBGT) values occasionally entering the “moderate risk” range despite the ostensibly benign seasonal climate (Havenga et al., 2024). Modelling further indicates that shifting the event to late winter (August) would substantially increase the likelihood of “very strong” WBGT-defined heat-stress conditions, underscoring the sensitivity of the race to seasonal timing and local microclimates (Havenga et al., 2022).

Heat stress during prolonged exercise increases core temperature, sweating rate, and cardio-vascular strain, thereby challenging thermoregulation and fluid balance (Périard et al., 2021). Ultra-endurance events held in tropical, desert, or otherwise exotic environments—particularly those requiring long-haul travel—further amplify heat-stress risk due to circadian disruption, altered hydration behavior, and unfamiliar climatic conditions (Bouscaren et al., 2019). For athletes preparing for such environments, heat acclimation consistently improves performance (Costa et al., 2014; Willmott et al., 2017), likely through adaptations that reduce cardiovascular and perceptual strain, enhance sweating efficiency, and improve thermal tolerance, collectively supporting greater exercise capacity in the heat (Tyler et al., 2024).

Extreme heat impairs all runners’ ability to perform in the *Western States Endurance Run*, but faster runners are at a greater disadvantage compared with slower competitors because they complete a greater proportion of the race in the hotter conditions (Parise and Hoffman, 2011). In a case study from the *Western States Endurance Run*, total energy expenditure was 16,104 kcal. Energy intake totaled 6,720 kcal (∼86 g carbohydrate/h). The athlete consumed 12.5 L of fluids (0.87 L/h; 18.5 g sodium) and lost 4.3% body mass. Mean gastrointestinal temperature was 37.1 °C and peaked at 39.4 °C. Pacing analysis showed a mean normalized speed of 84.8% of predicted critical speed, with a 15% decline across the race, demonstrating exceptional fatigue resistance (Mougin et al., 2026). In *Badwater*, the fastest finisher demonstrates a lower overall core body temperature. It may be possible that a time threshold exists whereby success in longer duration events requires an ability to maintain a lower core body temperature *versus* tolerating a higher core body temperature (Brown and Connolly, 2015).

Within the context of the *Comrades Marathon*, heat stress therefore represents a context-dependent but meaningful modifier of health and performance risk. While macro-climatic conditions suggest a generally low-risk environment, course-specific microclimates and moderate WBGT exposures can exacerbate dehydration, cardiovascular load, and renal stress in susceptible athletes (Havenga et al., 2024).

These findings highlight the importance of real-time environmental surveillance, individualized hydration strategies, and structured heat-acclimation protocols to mitigate risk during this uniquely demanding ultra-endurance event.

### Muscle cramping in the *Comrades Marathon*

Exercise-associated muscle cramping (EAMC) in *Comrades Marathon* runners is strongly associated with prior injury, chronic disease burden, and training-related factors, supporting a multifactorial and neuromuscular-fatigue–based etiology rather than a purely electrolyte-driven mechanism (Macmillan et al., 2024). These findings underscore the importance of optimized training-load management and appropriate medical screening in ultra-endurance athletes. The prevalence of EAMC in the *Comrades Marathon* is 3.2 per 1,000 race entrants (Sewry et al., 2022).

EAMC is characterized by involuntary, painful muscle contractions occurring during or immediately after exercise and has been linked to increased sweating, shifts in electrolyte concentrations, and altered neuromuscular control at the spinal level (Schwellnus, 2007). Management strategies therefore span several domains, including nutrition, hydration, exercise duration and intensity, sleep, and environmental conditions (Maughan and Shirreffs, 2019). In other endurance disciplines, the burden appears substantially higher. Among IRONMAN^®^ triathletes, the prevalence has been reported as 57.8 per 1,000 participants over three decades (Nilssen et al., 2024). Studies from the *Two Oceans Marathon* further highlight the complex risk profile. Chronic disease, medication use, a history of running injuries, and greater running experience were associated with EAMC (Schwellnus et al., 2018), while male sex, older age, longer race distances, specific training variables, allergies, and injuries within the preceding 12 months were identified as independent risk factors (de Jager et al., 2023).

Despite the prolonged mechanical loading inherent to ultra-distance and marathon running, most athletes experience only transient musculoskeletal effects. Post-race muscle and joint inflammation is common. However, imaging studies—including pre- and post-marathon MRI—have not demonstrated substantial structural knee damage in well-trained, asymptomatic runners (Fried, 2020). Nonetheless, athletes with pre-existing musculoskeletal conditions may experience symptom exacerbation, reinforcing the need for individualized risk assessment and targeted preventive strategies.

### Kidney injuries in the *Comrades Marathon*

Renal responses to ultra-endurance exercise span a continuum from normal physiological variation to clinically significant AKI. Distinguishing these categories is essential for interpreting post-race renal biomarkers and understanding the true incidence of pathological renal events in the *Comrades Marathon* and comparable ultradistance events.

#### Biological variations without clinical significance

A large proportion of renal perturbations observed during and after ultra-endurance running represent functional, adaptive responses rather than structural injury. Transient increases in serum creatinine, modest reductions in estimated glomerular filtration rate (eGFR), and mild hemoconcentration are common during prolonged exertion and largely reflect biological variation, altered renal hemodynamics, and increased creatinine production rather than true renal impairment. These changes typically normalize within hours to 24–48 h of rest and rehydration and should not be misclassified as AKI/acute renal impairment (ARI) (Fried, 2020).

Similarly, urinary biomarkers of tubular stress may rise after extreme endurance events without indicating structural injury. In the 2023 *Western States Endurance Run*, urinary concentrations of several AKI biomarkers—including IGFBP7·TIMP2—were elevated post-race, yet renal blood flow remained unchanged, supporting a functional rather than pathological mechanism. Euhydration appeared protective, attenuating biomarker increases (Linder et al., 2025).

Importantly, mild AKI observed in some ultra-marathoners does not appear cumulative. Runners with prior mild AKI did not demonstrate greater renal dysfunction during subsequent events of similar magnitude, indicating complete recovery and non-progressive physiology (Hoffman and Weiss, 2016).

#### Benign symptoms/events

A second tier consists of clinically mild, self-limited renal disturbances that may meet laboratory criteria for AKI but resolve rapidly and without sequelae. Mild AKI is common in events such as the *Western States Endurance Run*, where urine dipstick testing has shown excellent sensitivity and specificity for identifying runners meeting AKI criteria (Hoffman and Weiss, 2016). Despite meeting diagnostic thresholds, these cases typically normalize quickly and do not represent sustained renal injury.

Hydration status plays a central role. Hypohydration augments biomarker responses, whereas finishing euhydrated mitigates them. NSAID use—long implicated in exercise-associated renal dysfunction—remains a potential contributor, but recent reviews suggest limited evidence for clinically meaningful NSAID-related renal harm in young, healthy ultra-marathoners (Pannone and Abbott, 2024). Nonetheless, older athletes and those with comorbidities or polypharmacy exhibit greater susceptibility.

Exercise-associated rhabdomyolysis, another common finding in ultradistance events, rarely progresses to clinically significant renal impairment in healthy runners and typically remains within this benign category.

#### True AKI/ARI, including dialysis-requiring cases

The third tier comprises genuine pathological renal injury, which is rare but well documented in ultradistance events, including the *Comrades Marathon*. Historical reports from the 1970s describe several *Comrades runners* requiring hemodialysis or peritoneal dialysis due to ARF (MacSearraigh et al., 1979). More recent data from the *Western States Endurance Run* identified four runners over a three-year period who required prolonged dialysis (10–42 days) following severe AKI (Pasternak et al., 2023).

These cases reflect true clinical pathology, typically arising from the convergence of multiple stressors with exertional rhabdomyolysis, dehydration and hypovolemia, hypotension, hyponatremia or hypernatremia, hyperuricemia, and NSAID-related prostaglandin inhibition (Turgut et al., 2023).

NSAIDs are particularly relevant in this context. Prostaglandin-mediated vasodilation is essential for maintaining renal blood flow during dehydration, hypovolemia, and hypernatremia. NSAID-induced inhibition of prostaglandin synthesis can therefore precipitate renal hypoperfusion and clinically significant AKI in susceptible athletes (Gunaydin and Bilge, 2018).

True AKI is characterized by sustained creatinine elevation, reduced urine output, or evidence of tubular injury, and does not resolve with simple rehydration. Older runners and those with comorbidities or polypharmacy remain at disproportionately higher risk (Hörl, 2010).

Most renal perturbations observed in the *Comrades Marathon* represent reversible, functional physiology rather than structural injury. However, a small subset of athletes—particularly those exposed to compounding physiological or pharmacological stressors—may develop true AKI/ARI, including rare cases requiring dialysis. Recognizing the distinction between biological variation, benign transient AKI, and clinically significant renal injury is essential for accurate interpretation of post-race renal biomarkers and for guiding athlete management (Makris and Spanou, 2016).

### Strengths, limitations and future directions

A major strength of this review lies in its integrated and translational synthesis of more than five decades of scientific evidence derived from a single ultra-endurance event. By contextualizing performance trends, physiological responses, and medical outcomes within the unique characteristics of the *Comrades Marathon*, this review moves beyond descriptive reporting to provide clinically and practically relevant interpretation. The discussion systematically links epidemiological findings with underlying physiological mechanisms and medical risk factors, while highlighting how research conducted at the *Comrades Marathon* has directly contributed to advances in hydration strategies, medical screening, and race-day safety protocols. Moreover, the inclusion of large-scale prospective data alongside early foundational studies offers a rare longitudinal perspective on ultra-endurance running, reinforcing the *Comrades Marathon* as a robust natural model for studying extreme human performance and health outcomes.

Several limitations of the current evidence base warrant careful consideration. First, most available studies rely on observational, cross-sectional, or case-study designs, which inherently restrict causal inference. Longitudinal data, randomized comparisons, and standardized protocols are largely absent, limiting the ability to draw definitive conclusions about physiological mechanisms or health outcomes. Second, a substantial proportion of the medical and physiological literature originates from the SAFER research series. Although these studies are methodologically rigorous, they may overrepresent specific time periods, environmental conditions, and screening strategies, thereby constraining generalizability. Relatedly, many publications analyze overlapping cohorts of *Comrades Marathon* participants, particularly in biochemical and physiological investigations. This can lead to an overrepresentation of certain findings and complicates the interpretation of independent replication across studies. Third, female athletes remain markedly underrepresented, limiting sex-specific insights into performance, injury risk, and medical outcomes. This gap is especially relevant given known sex differences in endurance physiology, thermoregulation, and musculoskeletal injury patterns. Fourth, the literature spans several decades during which diagnostic criteria, medical protocols, laboratory methods, and race organization have evolved. Such temporal heterogeneity introduces variability that reduces comparability across studies and complicates trend analyzes. Fifth, there is considerable methodological heterogeneity across studies, including differences in sampling time points, biomarker panels, physiological testing protocols, and outcome definitions. Many investigations are exploratory and lack control groups, further reducing the strength of causal interpretations. Moreover, several commonly reported physiological and biochemical markers exhibit substantial acute post-race changes, yet their clinical significance remains uncertain. Elevations in these markers do not necessarily indicate pathology. In many cases, they likely reflect transient, adaptive responses to extreme endurance stress rather than adverse health outcomes. The literature does not consistently differentiate between findings supported by multiple independent studies and those based on single, isolated investigations. For example, participation trends, performance trajectories, and age-related patterns are well supported, whereas many physiological outcomes rely on limited evidence. Finally, long-term follow-up data are scarce. Few studies incorporate advanced imaging, continuous physiological monitoring, or multi-year health assessments, limiting insight into the chronic implications of repeated participation in the *Comrades Marathon*. Taken together, the current evidence base should be viewed as exploratory and descriptive rather than definitive. Future research should prioritize larger and more diverse samples, longitudinal designs, standardized methodologies, appropriate comparison groups, and comprehensive long-term monitoring to strengthen the robustness and applicability of findings.

Future research should prioritize prospective, multicenter study designs that employ standardized medical definitions, harmonized outcome measures, and uniform reporting frameworks to enhance comparability across race editions and across different ultra-endurance events. The current evidence base remains fragmented largely because studies vary widely in diagnostic criteria (e.g., for EAH, AKI, heat illness), timing of assessments, and follow-up duration. Establishing consensus methodological standards—analogous to CONSORT, STROBE, or IOC consensus statements—would substantially improve interpretability and meta-analytic potential.

Greater inclusion of female athletes, older age groups, and non-elite participants is essential to improve external validity. Most existing studies disproportionately sample male, middle-aged, high-performing runners, limiting generalizability and obscuring sex-specific or age-related risk profiles.

The *Comrades Marathon* represents a uniquely valuable natural laboratory for studying the longitudinal effects of repeated ultra-endurance exposure, particularly regarding renal function trajectories, cardiovascular remodeling, and musculoskeletal adaptation. Its annual format, large sample size, and consistent environmental context make it ideally suited for multi-year cohort designs capable of disentangling acute, cumulative, and adaptive physiological responses.

Integration of wearable technologies, continuous core-temperature monitoring, and serial biomarker profiling (renal stress markers, cardiac troponins, inflammatory mediators) may enable more precise risk stratification and earlier detection of adverse outcomes. These tools also offer opportunities to link real-time physiological load with post-race clinical findings, a connection that remains underexplored.

Finally, translating these insights into evidence-based guidelines for hydration strategies, medication use (particularly NSAIDs), training-load management, and pre-race medical screening remains a key priority for improving athlete safety.

## Conclusions

Completion of the *Comrades Marathon* is associated with a range of well-documented post-race physiological disturbances. Among the most distinctive findings are the unusually high rates of acute kidney injury and EAH, both of which carry important implications for athlete preparation, in-race monitoring, and post-race medical care during ultra-endurance competitions. Although several investigations have examined fluid balance and electrolyte regulation in *Comrades Marathon* runners, surprisingly little is known about their nutritional strategies or the specific metabolic demands imposed by this unique race. Key aspects of metabolic response—particularly substrate utilization, energy availability, and gastrointestinal tolerance—remain largely unexplored. Future research should address these metabolic dimensions to better inform evidence-based guidelines for training, race-day nutrition, and medical support. Advancing understanding in these areas will ultimately enhance athlete safety and performance in ultra-endurance running.

## Data Availability

The original contributions presented in the study are included in the article/supplementary material. Further inquiries can be directed to the corresponding author.
